# The basal ganglia select the expected sensory input used for predictive coding

**DOI:** 10.3389/fncom.2015.00119

**Published:** 2015-09-23

**Authors:** Brian Colder

**Affiliations:** Colder ScientificMcLean, VA, USA

**Keywords:** basal ganglia, predictive coding, action selection, cortical networks, emulation, top-down and bottom-up interaction, expectations, prediction

## Abstract

While considerable evidence supports the notion that lower-level interpretation of incoming sensory information is guided by top-down sensory expectations, less is known about the source of the sensory expectations or the mechanisms by which they are spread. Predictive coding theory proposes that sensory expectations flow down from higher-level association areas to lower-level sensory cortex. A separate theory of the role of prediction in cognition describes “emulations” as linked representations of potential actions and their associated expected sensation that are hypothesized to play an important role in many aspects of cognition. The expected sensations in active emulations are proposed to be the top-down expectation used in predictive coding. Representations of the potential action and expected sensation in emulations are claimed to be instantiated in distributed cortical networks. Combining predictive coding with emulations thus provides a theoretical link between the top-down expectations that guide sensory expectations and the cortical networks representing potential actions. Now moving to theories of action selection, the basal ganglia has long been proposed to select between potential actions by reducing inhibition to the cortical network instantiating the desired action plan. Integration of these isolated theories leads to the novel hypothesis that reduction in inhibition from the basal ganglia selects not just action plans, but entire emulations, including the sensory input expected to result from the action. Basal ganglia disinhibition is hypothesized to both initiate an action and also allow propagation of the action’s associated sensory expectation down towards primary sensory cortex. This is a novel proposal for the role of the basal ganglia in biasing perception by selecting the expected sensation, and initiating the top-down transmission of those expectations in predictive coding.

## Prediction in Perception

The importance of predictions in cognition has been extensively reviewed in recent neuroscience and cognitive science literature (Grush, 2004; Hawkins and Blakeslee, 2004; Friston and Stephan, 2007; Pezzulo et al., 2008; Bar, 2009; Bubic et al., 2010; Colder, 2011; Clark, 2013). Sensory perception is thought to result from the neural combination of “top-down” sensory expectations with “bottom-up” information from sensory organs (Bar, 2007; Panichello et al., 2013). In particular, the term “predictive coding” describes the theory that sensory expectations flow down from higher-level association areas to lower-level sensory cortex, and deviations from those expectations (error signals) flow back up to association areas (Rao and Ballard, 1999; Huang and Rao, 2011). Recent empirical evidence supporting predictive coding, (reviewed in Egner and Summerfield, 2013) include brain imaging results demonstrating increased activity in primary visual cortex in response to unexpected stimuli (Alink et al., 2010), and increases in the differentiation in the primary visual cortex responses to houses and faces as stimulus predictability decreases (Egner et al., 2010).

While the studies cited above, and others, support the notion that lower-level interpretation of incoming sensory information is guided by top-down sensory expectations, less is known about the source of the sensory expectations or the mechanisms by which they are spread. The “reafference principle” (von Holst and Mittelstaedt, 1950) states that copies of motor commands (“efference copies” or “corollary discharge”) are transmitted to sensory processing regions so that the sensation resulting from those actions (the “reafference”) can be “subtracted out” from the stream of incoming sensory information. Corollary discharge is used to anticipate and ignore the visual blur that occurs during high-speed saccades (Ross et al., 2001) and to inhibit the cricket’s auditory system response to self-generated noise (Poulet and Hedwig, 2006). Along with alerting the sensory system to very specific information that should be ignored or accounted for, corollary discharge may also provide more general information about potential actions that could be used to guide sensation.

Perception is an ongoing process, requiring a constant flow of top-down expectations to guide interpretation of incoming sensation. Action selection is also an ongoing process, and since action selection must consider predictions for the environment that would result from the action, each representation of a potential action must be tied to a sensory expectation. These action-dependent environmental predictions may serve as the top-down sensory expectations used in predictive coding.

## Link Between Potential Action and Expected Sensation

Prediction is a critical aspect of action planning and execution (for a review, see Mehta and Schaal, 2002). For instance, a constant downward force can produce the illusion of an increase in force if visual information leads to a prediction that the sensation of force should decrease (Diedrichsen et al., 2007). Also, trans-cranial magnetic stimulation over the cerebellum leads to reaching errors that suggest the cerebellum holds an estimate of future limb position (Miall et al., 2007). A classic theory of motor cortex states that motor cortex represents images of potential achievement, and continuously monitors progress toward those future goals (Pribram, 1971). The description of motor cortex function by Pribram (1971), based on anatomical and neurophysiological studies of the spinal cord, cerebellum, and motor cortex, emphasizes the similarities between neural representations of action plans and expected sensory states. Similarly, “common coding theory” (Prinz, 1997) explicitly states that perceived events and potential actions are represented in the same manner. Prinz (1997) also introduces the “action-effect” hypothesis, which holds that action planning depends on the expected outcomes of the potential actions. More recently, Friston suggests that motor intentions are tied to sensory predictions, and actions are designed to elicit sensory proprioceptive predictions (Friston, 2003, 2011). Clark (2013) describes this work as “action-oriented predictive processing”.

Further theoretical support for a strong link between planned actions and their expected sensation comes from Gross et al. (1999), who present a theory and model of generative perception based on the integration of action plans and their expected future sensory consequences. In addition, Grush (2004, 2007—skill theory v 2.0) introduces the “emulation framework” to explain how cognition might generate and use predictions of the sensory environment that would result from specific actions. As used here, the term “emulation” describes a single entity made up of the linked representations of a potential action and its expected sensory result. Empirical support for the link between potential actions and predicted outcome comes from a recent study that found anticipatory activity from single cell discharges in rat thalamus, and primary sensory and motor cortex, when rats explored the size of an aperture (Pais-Vieira et al., 2013). This anticipatory activity was disrupted when motor cortex was de-activated with muscimol, indicating that motor cortical activity was important for maintaining predictive representations in thalamus and sensory cortex.

The close ties between potential actions and the predictions for sensation those actions would produce led Colder (2011) to hypothesize that it was the sensory expectations in active emulations that were passed down to primary sensory cortices in the process of predictive coding. While Colder (2011) describes a theoretical source of top-down sensory expectations for perception, insight into how those expectations may be spread to lower-level cortices is still lacking. The link between potential actions and their predicted sensations suggests that the process of action selection may also be involved in spreading sensory expectations. To understand how action selection might spread predicted sensation, first we must consider how emulations are instantiated in neuronal networks.

## Parallel, Distributed Networks Instantiate Emulations

Fuster (2006, 2009), proposed that cortical representations of actions and sensory states are instantiated in “cognits”, or widely distributed neuronal networks. Emulations are hypothesized to be implemented in cognits consisting of neurons distributed widely throughout cortex (Colder, 2011). Similar to working memories (Fuster, 2006), emulations are hypothesized to be generated by partial sensory cues that activate neural networks associated with long-term memories. Emulations may begin as more abstract plans and more uncertain sensory expectations (e.g., considering whether to head to the park in the afternoon), in which case active cognits will include neurons from anterior portions of PFC, and higher-level sensory association areas, but few neurons from primary motor or sensory areas. Alternatively, if newly generated emulations include concrete, immediate plans and very specific sensory expectations (e.g., jumping out of the way of an oncoming bus), their initial active cognits will include primary sensory and motor regions.

**Figure 1:**
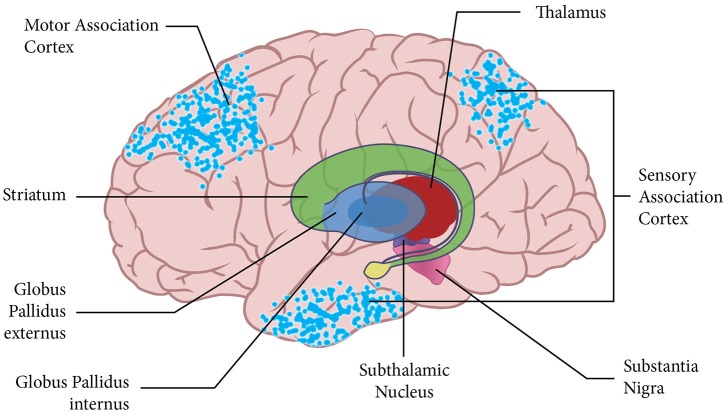
**The figure shows the cortical surface with an overlay of the basal ganglia and thalamus.** The blue dots on the cortical surface represent the neuronal activity for a notional emulation. This emulation is abstract, and the active neural networks instantiating the emulation are in higher-level motor and sensory association areas of the brain.

Figure 1 demonstrates the distributed nature of the active neuronal networks that instantiate emulations. The neural network for the notional abstract emulation depicted in the Figure includes neurons in frontal motor association areas whose activity corresponds to the emulation’s potential action. The emulation’s network also includes neurons in parietal and temporal sensory association areas whose activity corresponds to the expected sensory outcome of the potential action.

Many emulations may be simultaneously instantiated in parallel, distributed neural networks. This proposal follows the notion that association cortices are comprised of parallel, segregated networks as described by Goldman-Rakic (1988). Recent evidence supporting the idea of physically widespread neural networks as basic units of representation comes from a study of cortical connectivity finding interdigitated, functional networks distributed throughout the cortex (Yeo et al., 2011).

Continuous competition among emulations may be driven by incoming sensory information that defines the expected outcome of actions. In a visual discrimination task requiring saccades, activity in motor cortex reflects the continuous accumulation of sensory information that indicates the choice with best expected outcome (Gold and Shadlen, 2000). More recently, cortico-spinal excitability for action pathways was found to vary with the value of the action (Klein-Flügge and Bestmann, 2012). Incoming sensory information is important not only for updating the expected outcome of actions, but also for specifying which actions are physically possible at a given moment. Cisek (2007) proposes that incoming sensory information drives a continuous competition among potential actions, or “affordances”, by continuously updating the set of viable affordances.

Since emulations are instantiated in cortex as distributed neural networks, neural network operations govern the spread and shrinkage of emulation influence. In particular, spreading sensory expectations to lower-level sensory cortices requires activating lower-level sensory cortical neurons that are connected to the neurons instantiating the higher-level representation of the sensory expectation. Theories of action selection (discussed below) describe how the basal ganglia interact with cortical networks instantiating potential actions to allow the spread of the chosen network and action. The same mechanism can also serve to spread the network instantiating the sensory expectation associated with the action.

## Basal Ganglia Gate Actions from Within Cortical-BG-Thalamo-Cortical Information Loops

**Figure 2:**
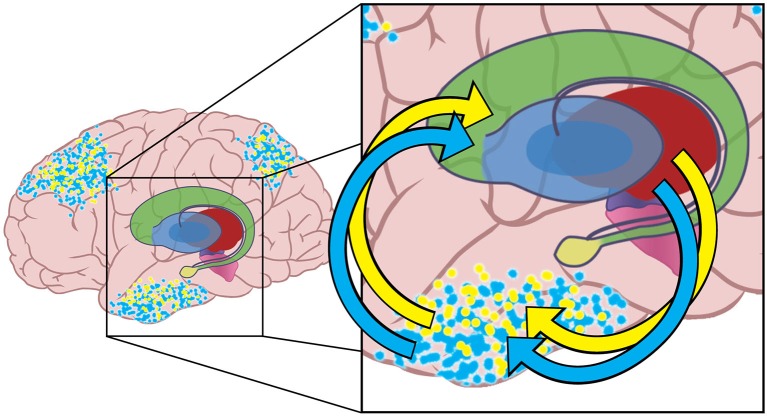
**The yellow dots represent activity in a neural network instantiating another notional emulation that also has activity in higher-level association areas of the brain.** The blue and yellow emulations contain different potential actions and expected outcomes. Multiple emulations are hypothesized to compete for realization at any given time.

Tract-tracing studies indicate that information flows from cortex through the basal ganglia and thalamus and back to cortex in mainly segregated loops (Alexander et al., 1986; Middleton and Strick, 2002) although there is some integration of information between loops (Haber and Calzavara, 2009). Although most empirical evidence demonstrates information loops between BG and PFC, retrograde labeling of the substantia nigra pars reticulata (SNr) reveals projections through thalamus to the inferotemporal cortex as well (Middleton and Strick, 1996), suggesting the existence of closed information loops from all parts of cortex through the BG and back to cortex. Figure 2 depicts the cortical-basal ganglia-thalamo-cortical loops for inferotemporal cortex for notional blue and yellow emulations.

The neural networks for both emulations have closed-loop connections with the basal ganglia and thalamus. The arrows shown indicate the excitatory input from one sensory association cortical region to the basal ganglia, and the excitatory path back from the thalamus to cortex. Information in these loops travels multiple pathways though the basal ganglia to reach thalamus and then return to cortex. Basal ganglia GO pathways increase excitation in cortical neural networks, while NoGo pathways decrease cortical excitation. Note that the neural networks in other cortical regions (e.g., pre-frontal cortex, and parietal sensory association areas) are also a part of similar cortical-basal ganglia- thalamic loops.

## Role of Cortical-BG-Thalamo-Cortical Information Loops in Action Gating

The BG play an important role in determining which of many possible actions might be performed (Mink, 1996; Redgrave et al., 1999). Mink (1996) hypothesized that once a movement is initiated in cortex, pathways from cortex through the BG decrease inhibition for the selected action, and increase inhibition on competing actions, allowing for the smooth execution of the selected action.

Whereas Mink (1996) and Redgrave et al. (1999) suggested that the BG select actions by disinhibiting the cortical portion of a cortical-BG-thalamo-cortical information loop containing an action plan, I propose that the information loops operate on not just action plans, but complete emulations, including the expected resulting sensation associated with action plans. Therefore the same mechanism hypothesized by Mink (1996) and Redgrave et al. (1999) to select actions is also selecting expected sensation.

## BG Go and NoGo Pathways

There are multiple pathways information could travel through the BG in a cortical-BG-thalamo-cortical information loop (Schroll and Hamker, 2013). For all pathways, the final connection, from thalamus to cortex, is excitatory. The preceding connection is inhibitory output from the BG [from either the globus pallidus internus (GPi) or SNr] to the thalamus. As a result, the BG provide a varying inhibitory influence on an excitatory connection from thalamus to cortex.

One of these pathways, the “direct” pathway through the BG includes an excitatory signal from the cortex to the striatum, which then sends an inhibitory signal to the BG output nuclei. As a result, activation of the direct pathway results in a decrease of BG output, producing less inhibition of the thalamus and cortex. The direct pathway was termed the “Go” pathway by Hazy et al. (2007). There are also multiple “NoGo” pathways through the BG. These pathways ultimately produce increases in inhibition from the BG to the thalamus, resulting in less cortical excitation (see review in Schroll and Hamker, 2013).

**Figure 3:**
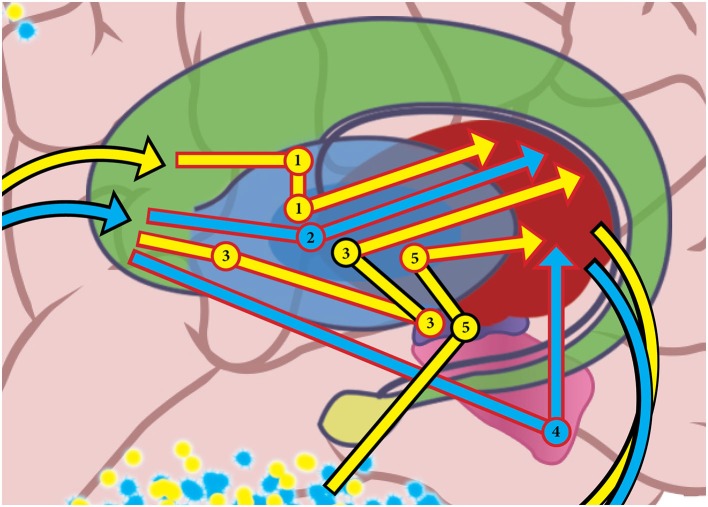
**In this figure, basal ganglia GO pathways are shown in blue, and they are selecting the “blue” emulation, while NO GO pathways are shown in yellow deselecting the “yellow” emulation.** Arrows that are outlined in black denote an excitatory connection, while red-outlined arrows represent an inhibitory connection. Pathway 1 is a NO GO pathway that includes an inhibitory connection from the striatum to the globus pallidus externus (GPe), an inhibitory connection from the GPe to the globus pallidus internus (GPi), and an inhibitory connection to the thalamus. Pathway 2 is a GO pathway that has an inhibitory connection from the striatum to the GPi, and an inhibitory connection from the GPi to the thalamus. Pathway 3 is a NO GO pathway that contains an inhibitory connection from the striatum to the GPe, an inhibitory connection from the GPe to the subthalamic nucleus (STn), an excitatory connection from STn to GPi, and an inhibitory connection from GPi to thalamus. Pathway 4 is a GO pathway that has an inhibitory connection from the striatum to the substantia nigra pars reticulata (SNr), and an inhibitory connection from SNr to thalamus. Instead of starting in the striatum, Pathway 5 is a NO GO pathway that includes an excitatory connection directly from cortex the STn, an excitatory connection from STn to GPi, and an inhibitory connection from GPi to thalamus. Note that the pathways shown are just a selected subset of all the possible pathways information can travel through the basal ganglia in a cortical-basal ganglia-thalamo-cortical loop. See Schroll and Hamker (2013) for a review of basal ganglia pathways.

Mink (1996) and Hazy et al. (2007) suggested that actions are selected when Go pathway activation for the cortico-BG-thalamo-cortical loop disinhibits the selected PFC action representation, at the same time as NoGo activity increases for competing actions in different information loops. Both Go and NoGo pathways were recently found to be active during normal movement (Cui et al., 2013), supporting the concept that action selection results from changes in the activation of each pathway relative to the other. Figure 3 shows some of the pathways information travels through the BG in cortical-BG-thalamo-cortical loops. Information about the blue emulation is moving on GO pathways, while the yellow emulation is being deselected by NO GO pathways.

## Proposal for Emulation Realization via Recruitment of “Actualization” Neurons

Following Mink (1996) and Hazy et al. (2007), I propose that a decrease in inhibition caused by excitation of BG Go pathway promotes selected emulations (potential action/expected sensation pairs) by increasing the firing rate in their instantiating neural network, while simultaneously BG NoGo pathways deliver increased cortical inhibition to competing emulations. Although there are many influences on cortical neural networks, this BG-produced relative increase in firing rate of the network for the action in the selected emulation allows the network to recruit more caudal/posterior neurons in PFC. This recruitment advances the activity in the selected action’s neural network toward the motor cortex and eventual execution. Thus, action selection results from BG disinhibition of emulations and their cortical networks that allows recruitment of more caudal/posterior PFC neurons, including motor strip neurons.

**Figure 4:**
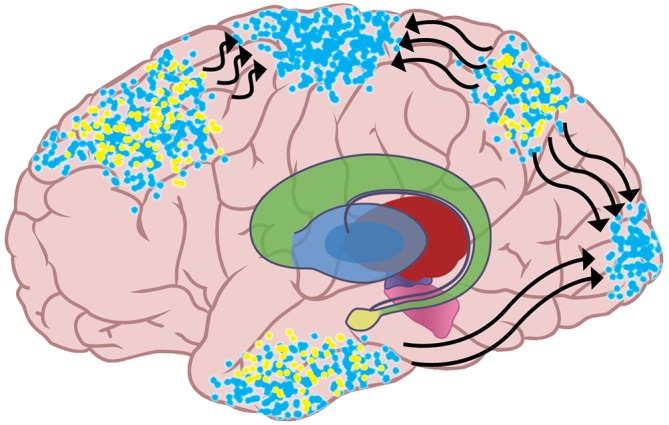
**The increased activity in the neural networks instantiating the blue emulation allows those neural networks to recruit neurons in lower-level sensory and motor areas.** The spreading of the emulation sends the action plan to the motor strip, which initiates the emulation’s potential action. The spread of the emulation to primary sensory cortices is the top-down propagation of the sensory expectations in the emulation, as described in predictive coding theory.

But since potential actions and expected sensations are linked, a decrease in inhibition for an emulation also increases the activation of the emulation’s posterior cortical network, allowing the network to recruit neurons that are physically closer to primary sensory cortices. This recruitment is the top-down dissemination of the emulation’s sensory expectations on lower-level sensory areas, and it is the implementation of predictive coding. BG disinhibition of preferred emulations and their distributed cortical networks is thus proposed to allow the spread of top-down expectations that shape ongoing perception. Figure 4 shows that since excitatory input from thalamus has increased for the blue emulation, the active neural networks for that emulation have recruited more neurons. Activity for the blue emulation has spread to primary motor cortex, meaning that the emulation’s action is being performed. Neural networks instantiating the blue emulation have also spread to primary somato-sensory cortex and primary visual cortex, indicating that the emulation’s sensory expectations are being used to interpret incoming sensation.

## Assumption that Cortical Networks Compete for Lower-Level Neurons

The theory described above depends on an important assumption about the spread of representations implemented in distributed cortical networks. While output from the basal ganglia is just one of many sources of excitation or inhibition for the neural networks instantiating emulations, the proposed mechanism for moving an emulation toward realization is that the emulation’s neuronal network is able to recruit lower-level neurons by virtue of its being disinhibited by the BG relative to competing emulations/networks. The assumption implicit in this mechanism is that the relative decrease in BG inhibition will increase the discharge rate of neurons in the distributed network, and that this increase in neuronal activity (compared to other networks) enhances the network’s ability to expand. This assumption will hold up in a scenario where lower-level sensory and motor neurons are constantly “in demand” by multiple cortical networks/emulations that are all competing to access lower-level neurons so that the emulations can “become reality”.

Note that action plans will become reality if neurons in primary motor cortex discharge in accord with the plan, causing the body to execute the action. The expected resulting sensory input might not always occur, but imposing expectations tends to bias perception toward the expected sensations (for reviews, see Summerfield and Egner, 2009; Seriès and Seitz, 2013). The importance of expectations in affecting perception was recently affirmed by a psychophysical study in which subjects tended to perceive random dot rotation in the expected direction, even when actual dot motion was completely ambiguous (Sterzer et al., 2008). In another task requiring subjects to press a button when a moving ball was at a specific location, subjects were found to consistently underestimate their own errors relative to errors they believed to be made by others (Wolpe et al., 2014). The authors interpret these findings as indicating that subjects had a prior expectation of successful task completion that biased their perception of their actual performance. These studies support the notion that a cortical network able to recruit lower-level sensory neurons would bias the organism towards experiencing the emulation’s sensory prediction as reality.

The idea that distributed networks based mainly in association cortex compete for neurons in lower-level sensory and motor areas is supported by imaging studies demonstrating increased activation in more anterior PFC as tasks became more complex (Koechlin et al., 2003; Badre and D’Esposito, 2007). Koechlin et al. (2003) explained these results using a “cascade model” of control in the PFC, in which representations at lower (more caudal) levels are controlled by representations at higher (more rostral) levels. Badre and D’Esposito (2007) also noted a general increase in baseline activity in more posterior areas of PFC when more anterior, higher-level areas become engaged. This increase in baseline in lower-level areas is consistent with the notion that abstract action plans implemented in networks that include anterior PFC compete for lower-level neurons in more posterior regions of PFC. Reviews by Fuster (2009) and O’Reilly (2010) conclude that action execution begins with abstract, complex representations in anterior PFC and proceeds to concrete representations and specific movements controlled by posterior PFC. Redgrave et al. (1999) also assumed that multiple action plans competed for access to motor effectors, and proposed the BG as the action plan selector.

## Novel Proposal for the Role of BG in Perception

The theory of BG function described above is drawn directly from hypotheses for the role of the BG in action selection (Mink, 1996; Redgrave et al., 1999; Hwang, 2013). Integrating these hypotheses with the notion that potential actions are bound to their expected resulting sensation, and also with theories of the role of expectations in perception suggests the novel role of the BG in also selecting and initiating top-down sensory expectations, and hence influencing perception.

Although BG disinhibition has not been previously suggested as the mechanism for propagating sensory expectations, there is considerable recent evidence suggesting that the BG play an important role in perception (reviewed in Ding and Gold, 2013). Single cell recordings from the caudate revealed the presence of neurons whose discharge rates tended to predict perceptual choices, especially when the sensory evidence for the choice was relatively weaker (Ding and Gold, 2010). In support of the current theory, these neurons appear to act like a source of top-down bias to lower-level sensory areas. Also, micro-stimulation of the caudate nucleus during a perceptual decision task induced a decision preference opposite to that preferred by neighboring caudate neurons (Ding and Gold, 2012). The authors use these results to suggest that the direct and indirect pathways could have opposite effects on perceptual decisions.

Multiple authors have previously modeled BG computations involving perception. Rao (2010) describes a model in which the BG select actions based on previously learned responses to Bayesian belief states represented in cortex. The belief states and learned actions described by Rao (2010) are similar to emulations. Bogacz and Gurney (2007) and Lepora and Gurney (2012) suggest that the BG optimally select actions based on associated perceptual hypotheses. The associations of actions and perceptual hypotheses used in those models are also similar to emulations.

## General Discussion

I propose that the internal Go and NoGo pathways of the BG select between multiple alternate futures (emulations) that are implemented in distributed cortical networks and cortical-BG-thalamo-cortical information loops. The result of this selection is the disinhibition of selected cortical networks, allowing them to recruit lower-level sensory and motor neurons, which has the effect of advancing the selected emulation to actualization, including spreading the emulation’s sensory expectation. Although previous authors have made similar proposals to describe how actions are selected, a novel aspect of this proposal is that BG disinhibition initiates the spread of top-down sensory expectations that are known to play an important role in perception. The hypothesis depends on the assumption that the neural networks instantiating emulations compete for access to lower-level neurons that can implement the emulation’s potential action and sensory expectations.

This hypothesis derives from previous research and theory indicating the role of the BG’s Go and NoGo pathways in action selection, combined with another body of literature describing the importance of prediction in cognition, including predictive coding in perception. Extending the BG’s role in action selection to a more general role for selecting between alternate futures follows naturally from the notion that action plan representations are linked to representations of the resulting expected sensation to create complete future scenarios. This assumption agrees with Pribram (1971), who suggested that action plan representations are just the expected sensory outcome, including the state of muscle spindle cells, after the action has been performed. In this view, also shared by Friston (2003, 2011), action planning is based on sensory representations, and a sequence of actions can be conceived as a series of expected sensations that include signals of muscle position. The present hypothesis goes beyond these theories relating potential actions and expected sensation, as well as Prinz (1997)’s common coding theory, by presenting a candidate mechanism, BG disinhibition, for the selection of actions and the propagation of expectations.

The hypothesis that action selection occurs as BG-disinhibited cortical networks recruit neurons closer to primary motor cortex stands in contrast to the proposal from Rizzolatti and Luppino (2001) that prefrontal “control areas” with a predominance of prefrontal connections determine when activity in more parietal-connected, posterior frontal cortex leads to action. Perhaps these hypotheses are in agreement if cortical-BG-cortical networks that originate from prefrontal “control areas” are initially disinhibited, leading to recruitment of more posterior prefrontal networks.

## BG Role in Predicting Reward

Evidence that the BG track the outcomes of actions—including reward information—supports the hypothesis that the BG select emulations that include expected sensation. The BG are known to encode reward prediction errors (O’Doherty et al., 2004). Individual anterior caudate neurons encode both reward and task information (Yanike and Ferrera, 2014). Neurons in the caudate, putamen and ventral striatum showed activity related to the magnitude of the expected reward (Cromwell and Schultz, 2003). Also, caudate neurons have been found that are sensitive to the size of the expected reward and their output may produce a bias in the superior colliculus that influences saccadic output (Hikosaka et al., 2006). More recently, multiple, distinct relationships between reward size and baseline neuronal discharge characteristics were found in recordings from neurons throughout the caudate (Nakamura et al., 2012).

From the perspective of the current hypothesis, reward is an important part of the more general expected sensation resulting from an action plan. Ding and Gold (2013) speculate that expectations of reward might be combined with other anticipated sensory information to aid in perceptual decisions. Exact mechanisms of how reward and other aspects of the expected sensory environment influence the BG’s Go and NoGo pathway calculations of inhibition for individual emulations and cortical-BG-thalamo-cortical information loops are yet to be determined.

## Potential Changes to Computational Models of BG Function

Previous models focused on the role of the BG in action selection (Hazy et al., 2007), gating of working memory (Frank et al., 2001) and the reinforcement learning needed to guide action selection (Hazy et al., 2006; Rao, 2010; Bolado-Gomez and Gurney, 2013). Other research has included modeling BG and cortex performance on perceptual decisions using models that assume sensory evidence is passively accumulated until a decision is reached (Bogacz and Gurney, 2007; Lepora and Gurney, 2012). The hypothesis that the BG initiate top-down sensory expectations suggests a novel addition to models of BG function, that BG disinhibition should encourage selected perceptual hypotheses before all sensory evidence is obtained, creating a bias that tends to influence perception towards those hypotheses. Thus, instead of passively accumulating evidence, models of the BG’s role in perceptual decisions might include terms that accelerate progress towards a specific decision after evidence supporting that decision has been observed.

## Testing the Hypothesis

The proposed hypothesis incorporates established theories of action selection by the BG, while emphasizing the link between representations of action plans and the anticipated sensation resulting from those plans. A large body of empirical evidence already exists describing how BG activity is linked to action selection. Therefore key tests of this hypothesis would shed light on the BG’s role in establishing sensory expectations. Informative experiments could manipulate the Go and NoGo pathways in the BG and measure the resulting effect on sensory expectations. For instance, there are two different NoGo pathways that pass through the subthalamic nucleus (STn; Schroll and Hamker, 2013), the indirect pathway [cortex to striatum to globus pallidus externus (GPe) to STn to GPi], and the hyperdirect pathway (cortex to STn to GPi). Reducing the effectiveness of the STn by electrical, chemical or mechanical means during the presentation of informative stimuli should disrupt these NoGo pathways. According to the proposed hypothesis, this disruption would result in a lack of inhibition for competing actions and expectations, which will decrease the top-down guidance for interpretation of incoming sensory stimuli.

Physiological measurements can provide indirect evidence of sensory expectations. For instance, Summerfield et al. (2006) found fMRI activity that corresponded to specific “perceptual sets” in a visual recognition task with degraded stimuli, and many studies have demonstrated modulation of cortical single neuronal firing patterns by anticipated reward (for example, Rosenkilde et al., 1981; Shuler and Bear, 2006; Burton et al., 2013; Stanişor et al., 2013). If BG disinhibition is responsible for spreading sensory expectations as described above, then inactivating the BG should reduce the neurophysiological changes corresponding to lower-level sensory expectations, including reward expectations. That is, while “reward cells” may still be found in pre-frontal cortex and sensory association cortices, it should not be possible to observe reward cells in primary sensory and motor cortices when the BG are inactivated. Alternatively, if the activity of just the STn of the BG was experimentally decreased, then the effectiveness of the NoGo pathway should be degraded, allowing incorrect expectations to propagate. In this case it may be possible to see reward cell activity even when rewards are not expected.

The degree to which an individual’s sensory expectations match incoming stimuli can also be inferred using behavioral measures such as reaction time or percent correct responses. However, it is difficult to obtain behavioral data on sensory expectations that is not confounded with selected actions—both a misperception and an incorrectly selected action will produce the same result, an incorrect choice. Specific tests of the proposed role of the BG in selecting and advancing potential sensation (as opposed to selecting action plans) must dissociate selection of sensory expectations from selection of an action plan. Perhaps this could be accomplished in a behavioral experiment by requiring the same response for all stimuli and measuring how changes in reaction time vary with manipulations of sensory expectations and BG function.

The experiments described above suggest the use of laboratory methods to manipulate BG function. Further evidence bearing on the hypothesis could come from “natural lesion” studies of patients with BG dysfunction that affects the motor system, such as Parkinson’s or diseases producing chorea. Since the current hypothesis supposes that the BG are involved in propagating sensory expectations, patients with BG dysfunction might be expected to show perceptual problems related to inaccurate sensory predictions. In fact, in an experiment on attentional set shifting, Cools et al. (2010) found that patients with Parkinson’s disease relied more on bottom-up stimulus characteristics—and less on top-down expectations—than did normal controls. Further testing is needed to determine if a reduced emphasis on top-down sensory expectation is a common feature of patients with BG-related disease.

## Application of the Emulation Framework

Colder (2011) proposed a theory of prediction in cognition that provided a general emulation-based framework for understanding cognitive neuroscience research. Placing studies of the BG into the emulation framework supports the current re-interpretation of findings that have previously been used to link the BG to action selection, resulting in the more general theory that the BG select between alternate potential futures. This is an example of how the emulation framework can offer a context for cognitive neuroscience research that may provide additional insight into the implications of research results.

## Conflict of Interest Statement

The author declares that the research was conducted in the absence of any commercial or financial relationships that could be construed as a potential conflict of interest.
